# Improving diagnostic interpretability of abdominal ultrasound for neonates with suspected intestinal injury

**DOI:** 10.3389/fped.2025.1677655

**Published:** 2025-10-23

**Authors:** Yogen Singh, Trevor Bushong, Eric Diaz, Elim Man, Belinda Chan

**Affiliations:** ^1^ Neonatology Division, Department of Pediatrics, University of California Davis Health Children’s Hospital, Sacramento, CA, United States; ^2^ Department of Radiology, University of California Davis Health Children’s Hospital, Sacramento, CA, United States; ^3^ Neonatology Division, Department of Pediatrics, Hong Kong Children’s Hospital, Kowloon Bay, Hong Kong SAR, China

**Keywords:** necrotizing enterocolitis (NEC), spontaneous intestinal perforation (SIP), malrotation, small bowel obstruction, exploratory laparotomy, abdominal ultrasound, transducer frequency, preterm infants

## Abstract

**Background:**

Abdominal ultrasound (AUS) is increasingly utilized as a diagnostic adjunct in neonates undergoing evaluation for intestinal injuries such as necrotizing enterocolitis (NEC), spontaneous intestinal perforation (SIP), volvulus, and intestinal obstruction, which need urgent surgical evaluation and often emergent intervention. However, the interpretability of AUS—defined as the number of explicit documentations of high-risk ultrasound findings (HRF)—varies in radiology reports, potentially influenced by clinical and technical factors.

**Objective:**

To identify clinical and technical factors associated with increased interpretability of neonatal AUS in the evaluation of suspected intestinal injury needing surgical intervention.

**Methods:**

This retrospective, single-center case series reviewed AUS exams performed from 2022 to 2024 at a level IV neonatal intensive care unit. All neonates who had AUS performed prior to exploratory laparotomy were included in the study. For this project “interpretability of AUS” was defined as the number of explicit reporting of eight predefined HRF indicative of surgical need: pneumoperitoneum, increased or decreased bowel wall thickness, reduced intestinal perfusion on color Doppler, absent or decreased peristalsis, bowel dilation, complex intra-abdominal fluid collections, and reversed orientation of the superior mesenteric artery and vein. Clinical and technical factors that may have potentially influenced interpretability were analyzed.

**Results:**

Twenty-eight AUS exams from 18 neonates were analyzed. The median gestational age at birth was 34^+2^ weeks, and the median birth weight was 1.93 kg. The median HRF of all AUS exams were 2 (range 0–8). Higher-frequency ultrasound transducers (>10 MHz) improved interpretability of AUS images (HRF 4 vs. 1), particularly in neonates weighing <2 kg. Serial ultrasound evaluations within seven days of surgery were associated with greater interpretability compared to a single isolated exam (HRF 6 vs. 3). Clinical symptoms with hypotension or abdominal discoloration and examinations ordered with comprehensive clinical details for the attention of radiology team showed trends towards improved interpretability.

**Conclusions:**

In our pilot study, interpretability of neonatal AUS images was strongly influenced by using higher-frequency transducers (>10 MHz) with better resolution, particularly in neonates weighing <2 kg. Obtaining serial imaging improved subsequent interpretability.

## Introduction

Abdominal ultrasound (AUS) has emerged as an essential imaging modality for evaluating neonates with suspected abdominal surgical emergencies, including necrotizing enterocolitis (NEC), spontaneous intestinal perforation (SIP), volvulus, and intestinal obstruction (1–8). Timely identification of these time-critical conditions is crucial, as delays in diagnosis are associated with increased morbidity and mortality. While abdominal x-ray (AXR) remains the primary imaging modality for assessment of abdominal pathology, AUS has proved to be an excellent adjunct. Previous studies have shown good agreement between x-ray and AUS (9–11). Combining both modalities can decrease time to diagnosis, especially for early NEC and complications like sealed perforation (9). In situations when initial x-rays are equivocal, AUS can provide additional markers of intestinal injury such as peristalsis, free peritoneal fluid including characterization of complex vs. simple fluid collections, quantitative assessment of intestinal wall thickness, perfusion with color doppler, and loss of intestinal wall signature (i.e., echogenic bowel wall) (10, 11).

The ultrasound findings associated with increased risk of surgical intervention include pneumoperitoneum, abnormal bowel wall thickness, echogenic bowel wall, absent bowel wall perfusion, loculated or complex fluid collections, reversal of superior mesenteric artery (SMA) and superior mesenteric vein (SMV) orientation, and decreased or absent bowel peristalsis (1–4, 7, 8, 11, 12). In agreement with the specialist radiologist, decreased bowel peristalsis was defined as segments of bowel with less than 5 contraction waves per minute after an observation time of at least 1 min (13). Although pneumatosis intestinalis and portal venous gas are classic radiographic findings of medical NEC, they have not been found to be strong predictors for surgical intervention (14). Accurate reporting of the presence or absence of these findings provides valuable information to both the primary clinician regarding the need for surgical consultation as well as the pediatric surgeon regarding the timing of surgical interventions.

Despite its utility in risk stratification for neonates with suspected abdominal emergencies, AUS remains difficult to implement in clinical practice. This is largely due to lack of availability of experienced personnel who can reliably obtain and interpret AUS imaging in neonates (15–17). Patient factors, such as presence of gaseous dilation of bowel loops which limits sonographic windows, can also lead to difficult or non-diagnostic exams because of non-interpretable imaging quality or inability to scan the whole intestine systematically (18). It is well known that ultrasonography evaluation is highly user and technical abilities dependent, but there is limited information regarding the barriers affecting the utility or yield of AUS for suspected intestinal injury.

This study aims to address this knowledge gap by identifying the barriers, both clinical and technical, affecting the interpretability of AUS in neonates with intestinal pathology requiring surgical intervention, and to identify factors that could be easily addressed through future quality improvement initiatives. To address the identified knowledge gap, we investigated the relationships between patient demographics, ultrasound transducer characteristics (including frequency), quality of clinical order indications, clinical presentations, and the practice of serial vs. single ultrasound examinations in influencing the number of high-risk findings explicitly reported. Improved understanding of these relationships may guide clinical practice and radiologic protocols, ultimately enhancing diagnostic accuracy and optimizing patient outcomes in the neonatal intensive care unit (NICU).

## Methods

### Study design and setting

We performed a single center retrospective case series observational study at a level IV NICU between Jan 2022 and Dec 2024, analyzing AUS examinations performed in neonates undergoing evaluation for suspected intestinal injury who subsequently required exploratory laparotomy. The study was approved by the institutional review board, and informed consent was waived due to the retrospective nature and de-identified data usage.

### Study population and inclusion criteria

**Figure 1:**
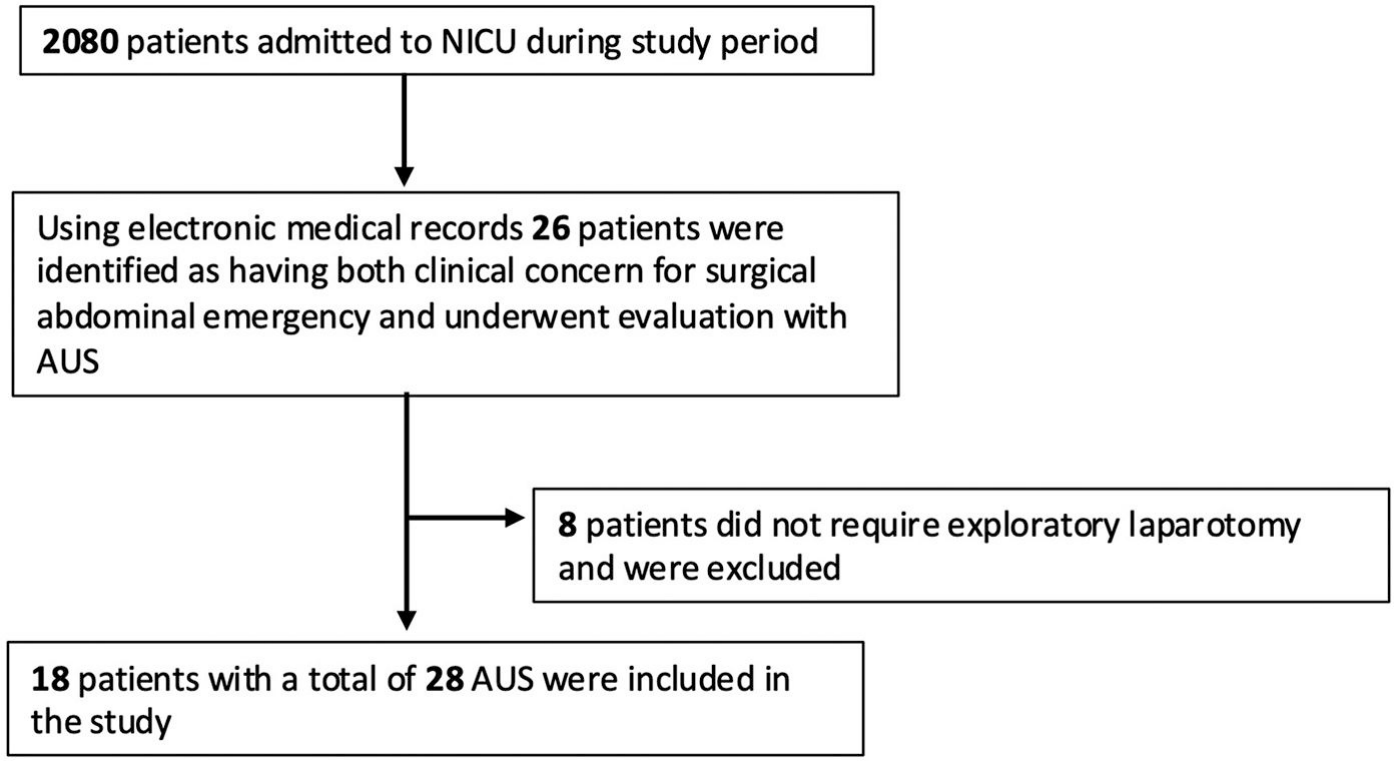
Flow diagram of patient selection. AUS, abdominal ultrasound.

All neonates who underwent exploratory laparotomy and had AUS performed prior to surgery were included. Eligible neonates were identified through surgical and radiology databases. Patients were included if they had at least one documented AUS obtained by a diagnostic medical sonographer with views of all four abdominal quadrants prior to surgery. A total of 2080 patients were admitted during the study period. Using our electronic medical record databases, 26 patients were identified as having both clinical concern for surgical abdominal emergency and underwent evaluation with AUS. Of those, 8 did not require exploratory laparotomy and were excluded. In total, 18 patients with a combined 28 AUS were included in the study (Figure 1). All AUS were formally interpreted by a radiologist. Exclusion criteria included AUS obtained in patients lacking clinical suspicion or diagnosis of intestinal injury, and neonates who did not undergo exploratory laparotomy or lacked surgical pathology to confirm the final diagnosis.

### AUS scanning protocol

At our institution, we developed a standardized protocol for assessing intestinal injury using AUS performed by diagnostic medical sonographers. Notably, this protocol was revised during the study period. Current Abdominal Ultrasound Protocol for Intestinal Assessment:

• Brief Abdominal Survey
  ◦ Evaluate for ascites (simple vs. complex)
  ◦ Evaluate for intraabdominal free air
• Imaging acquisition should include:
  ◦ Sweep cine clips
  ◦ Static images
  ◦ Static images with color Doppler
• Liver Evaluation
  ◦ Perform a complete grayscale sweep of the liver.
• Portal Venous Gas Assessment
  ◦ Acquire a cine clip at the main portal vein with the probe held still to assess for intraluminal air bubbles.
  ◦ If gas is detected, examine the peripheral liver for additional echogenic foci.
• Mesenteric Vessel Evaluation
  ◦ Assess the anatomical relationship between the superior mesenteric artery (SMA) and superior mesenteric vein (SMV).
  ◦ Evaluate for vascular swirling suggestive of volvulus.
• Bowel Assessment (all four quadrants)
  ◦ Assess peristalsis (<5 contraction per minutes)
  ◦ Assess for dilated bowel loops.
  ◦ Evaluate bowel wall echotexture, thickness, and presence of pneumatosis intestinalis.
  ◦ Evaluate for bowel wall perfusion with color Doppler

### Definition of interpretability

Interpretability was defined as the number of high-risk ultrasound findings (HRF) explicitly addressed in the radiologists’ reports, whether documented as present or absent. This definition was adapted from prior work on report clarity and completeness in NEC imaging which demonstrated that the degree of certainty with which key findings are reported can influence interpretability and clinical decision-making (19). Our study team (radiologists and neonatologists) agreed on the following HRF to be included:

• Pneumoperitoneum
• Increased bowel wall thickness
• Decreased bowel wall thickness
• Echogenic bowel wall
• Decreased intestinal perfusion as seen on color Doppler
• Decreased or absent peristalsis
• Bowel dilation
• Complex or loculated intraabdominal fluid collections
• Reversed orientation of superior mesenteric artery (SMA) and superior mesenteric vein (SMV) suggesting the presence of malrotation.

Each ultrasound report was retrospectively reviewed to quantify the total number of explicitly documented HRF. These findings were chosen due to their known association with the need for surgical intervention. Findings such as pneumatosis and/or portal venous gas can indicate high risk for intestinal injury on abdominal x-ray. In AUS, however, current evidence indicates that the presence of pneumatosis and/or portal venous gas does not correlate strongly with the need for surgical intervention when compared with other high-risk findings (HRF) on AUS (1–4, 7, 8, 20). These findings reflect disease activity but do not reliably predict transmural necrosis or perforation. Instead, surgical intervention is typically guided by clinical evidence of intestinal perforation (e.g., pneumoperitoneum) or severe, refractory disease. For this reason, pneumatosis and portal venous gas were not included as HRF in our study.

### Data collection

The following data were abstracted from electronic medical records and radiology reports:

• Patient Demographics: Gestational age at birth, birth weight, and weight at the time of ultrasound examination.
• Clinical Data: Presence or absence of hypotension and/or abdominal discoloration.
• Surgical Data: Date of exploratory laparotomy, surgical pathology diagnosis, and clinical outcome (survival or mortality).
• Ultrasound Technical Data: Transducer frequency [categorized as ≤10 MHz (defined as low frequency transducer) or >10 MHz defined as high frequency transducer] and transducer type.
• Ultrasound Examination Protocol: Whether the examination was part of serial imaging or a single isolated evaluation.
• Order Indications: Quality and specificity of the clinical indication provided to the reading radiologist at the time of ultrasound ordering. Order indications were categorized as “High Quality” or “Low Quality”. Orders with high quality included both detailed descriptions of the relevant patient history and clinical concern for intestinal pathology (21, 22).

### Statistical analysis

Descriptive statistics were calculated for patient characteristics and interpretability. Variables were summarized using medians with interquartile ranges. The “interpretability of AUS images”—defined as the number of HRF explicitly reported in radiology interpretations—was compared across categorical variables including transducer frequency, clinical signs, serial vs. single ultrasound exams, and quality of order indication.

Some patients underwent multiple abdominal ultrasound examinations, introducing dependence due to repeated measures. Although a mixed-model regression or repeated measures of non-parametric analysis would have been the appropriate method to assess statistical significance, this approach was not feasible given the small sample size. In addition, the HRF data were not normally distributed. Therefore, variables were mainly compared descriptively (e.g., high vs. low transducer frequency, presence vs. absence of clinical signs, serial vs. single examinations, and high- vs. low-quality order indications) rather than through detailed statistical analyses. For independent group comparisons, when median differences exceeded three, the Mann–Whitney *U* test was used to assess differences in diagnostic yield, acknowledging the limitations of the *p*-value obtained due to non-independence from repeated measures. A two-sided *p*-value < 0.05 was considered statistically significant. All analyses were conducted using Python (version 3.x), utilizing the SciPy and Pandas packages.

### Ethical considerations

The study received institutional review board approval (IRB-ID: 2335535-1), and patient data were anonymized prior to analysis.

## Results

### Study population

A total of 28 abdominal ultrasound (AUS) examinations were performed in 18 neonates who subsequently underwent exploratory laparotomy (Figure 1). The median gestational age at birth was 34 weeks and 2 days (IQR: 26 w 0 d–37 w 3 d). Birth weights ranged from 0.45 kg to 3.89 kg with a median of 1.93 kg (IQR 0.62–3.19 kg). The median weight at the time of AUS was 1.96 kg (IQR1.13–6.8 kg). Surgical diagnoses included NEC, SIP, gastroschisis, malrotation, volvulus, intestinal atresia, and intestinal obstruction. Four out of 18 neonates (44%) died prior to discharge (Table 1).

**Table 1:** Demographic characteristics and clinical outcomes of the neonates.

Patient ID	Birth gestational age	Birth weight (kg)	Clinical history	Clinical outcome
1	22w1d	0.45	SIP	Death
2	23w3d	0.53	SIP	Death
3	23w3d	0.50	Bowel perforation, hemoperitoneum	Death
4	25w4d	0.82	SIP	DC home
5	26w0d	0.79	Surgical NEC with ischemic bowel	DC home
6	26w1d	0.55	Initial concern for NEC, found to have malrotation with volvulus and catastrophic bowel injury	Death
7	30w3d	0.57	Surgical NEC with ischemic bowel	DC home
8	34w1d	1.29	History of gastroschisis who developed medical NEC after initial repair and found to have SBO requiring lysis of adhesion and bowel resection	DC home
9	34w3d	1.64	Surgical NEC with ischemic bowel	DC home
10	35w6d	2.42	History of Jejunal atresia who developed SBO after initial repair requiring resection of necrotic bowel	DC home
11	36w6d	3.20	Meconium pseudocyst with perforation	DC home
12	37w1d	2.22	History of Gastroschisis with malrotation who developed intestinal perforation and intrabdominal abscess after initial repair	DC home
13	37w2d	3.56	Meconium pseudocyst with *in utero* perforation	DC home
14	37w3d	3.42	Jejunal atresia	DC home
15	38w0d	3.16	Right sided CDH with malrotation who developed SBO after initial repair requiring lysis of adhesions	DC home
16	38w1d	2.98	Jejunal atresia	DC home
17	39w0d	3.89	Right sided CDH with coarctation, who developed surgical NEC with ischemic bowel after initial repair and requiring bowel resection	DC home
18	39w2d	3.41	Ileal atresia	DC home

DC, discharge; CDH, congenital diaphragmatic hernia; NEC, necrotizing enterocolitis; SBO, small bowel obstruction; SIP, spontaneous intestinal perforation; SMA, superior mesenteric artery; SMV, superior mesenteric vein.

### Interpretability

**Figure 2:**
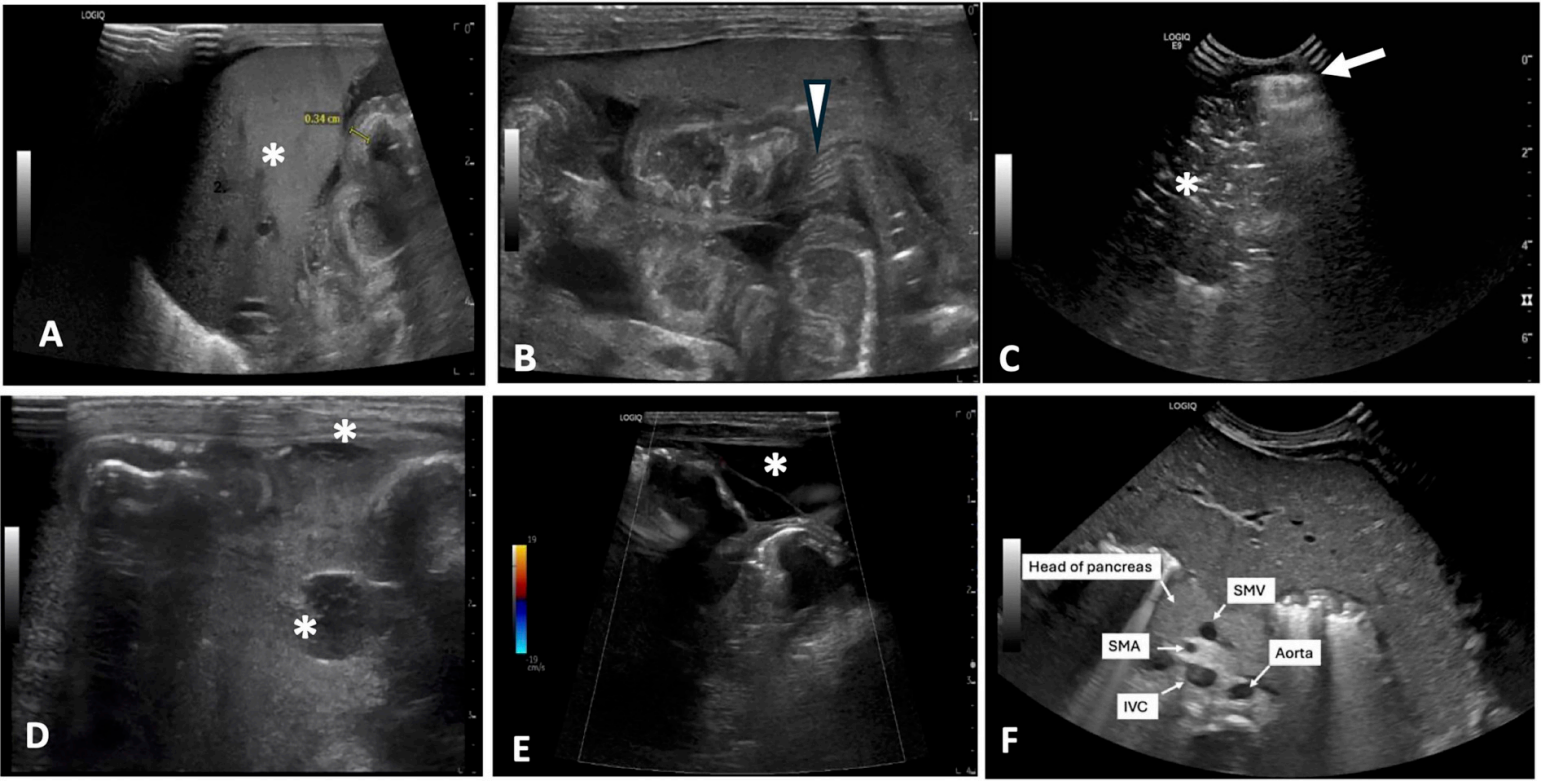
Representative AUS images of select HRF. **(A)** Thickened, echogenic bowel measuring 0.34 cm near liver (*). **(B)** Echogenic bowel wall with thickening of bowel valvulae conniventes and their hypoechoic interspaces resembling zebra stripe pattern (arrowhead). **(C)** Pneumoperitoneum seen as a bright echogenic stripe with reverberation artifact (arrow) just above anterior aspect of liver. Portal venous gas is also present (*). **(D)** Complex fluid collections (*). **(E)** Segment of bowel with diminished perfusion as seen on color Doppler imaging. Free fluid with septation is also seen surrounding bowel (*). **(F)** Reversal of SMV/SMA orientation with the SMV positioned to the left of the SMA. AUS, abdominal ultrasound; HRF, high risk findings; SMV, superior mesenteric vein; SMA, superior mesenteric artery; IVC, inferior vena cava.

Representative AUS images of select HRF are shown in Figures 2A–F. The technical and clinical variables affecting the interpretability of each exam are summarized in Table 2. The interpretability of each AUS exam had a median number of HRF of 2 (IQR: 1–5), ranging from 0 to 8 HRF per exam. Six AUS exams from four neonates at the beginning of the study period lacked documentation on all eight HRFs; consequently, no positive HRFs were identified, despite all four neonates having confirmed bowel perforation on surgical pathology. The lack of documentation—whether due to missing details in the radiology report or absent in images —made it unclear whether the high-risk findings (HRF) were truly negative or simply not evaluated. After these initial AUS studies, our unit standardized AUS image acquisition and documentation, leading to gradual improvement in HRF documented, even the negative findings were reported.

**Table 2:** Clinical and technical factors associated with all studied abdominal ultrasound examinations.

Patient ID	AUS exam ID	Transducer frequency (Hz)	Probe type	High risk clinical signs	Serial vs. single AUS	Weight at the time of AUS (kg)	High quality order indication	Days between AUS and ex lap	Number of HRF commented on	Number of positive HRF
1	1	10	Microconvex	Abdominal discoloration and hypotension	Single	0.42	No	2	0	0
1	2	10	Microconvex	Abdominal discoloration and hypotension	Single	0.50	Yes	0	3	3
1	3	10	Microconvex	Abdominal discoloration and hypotension	Single	1.15	Yes	0	2	2
2	4	15	Linear	Abdominal discoloration and hypotension	Serial	0.49	Yes	1	1	0
2	5	10	Microconvex	Abdominal discoloration and hypotension	Serial	0.50	No	2	0	0
3	6	10	Microconvex	Abdominal discoloration and hypotension	Single	0.93	Yes	0	0	0
4	7	15	Linear	Abdominal discoloration	Serial	1.07	Yes	2	6	0
4	8	9	Linear	Abdominal discoloration	Serial	1.07	Yes	0	2	1
5	9	18	Linear	Abdominal discoloration	Serial	1.20	No	1	7	3
5	10	20	Linear	Abdominal discoloration	Serial	1.20	No	0	8	5
6	11	15	Linear	Abdominal discoloration	Single	1.36	No	0	4	3
7	12	10	Microconvex	Neither	Single	1.81	No	3	1	1
8	13	15	Linear	Abdominal discoloration	Serial	1.96	Yes	12	8	3
8	14	15	Linear	Abdominal discoloration	Serial	1.96	Yes	11	8	3
8	15	15	Linear	Abdominal discoloration	Serial	1.96	Yes	5	7	3
8	16	15	Linear	Abdominal discoloration	Serial	2.71	No	2	2	2
8	17	15	Linear	Abdominal Discoloration	Serial	2.71	No	0	7	4
9	18	15	Linear	Neither	Single	2.70	No	0	4	2
10	19	15	Linear	Abdominal discoloration	Single	2.72	Yes	4	4	4
11	20	10	Microconvex	Neither	Single	3.19	No	1	1	1
12	21	9	Linear	Abdominal discoloration	Single	3.20	No	0	0	0
13	22	10	Microconvex	Neither	Single	3.24	No	1	2	2
14	23	9	Linear	Abdominal discoloration	Serial	3.48	No	1	1	1
14	24	15	Linear	Abdominal discoloration	Serial	3.48	No	0	2	2
15	25	10	Microconvex	Abdominal discoloration	Single	3.56	No	1	0	0
16	26	15	Linear	Neither	Single	3.64	Yes	4	3	2
17	27	15	Linear	Neither	Single	4.03	No	1	0	0
18	28	9	Linear	Hypotension	Single	6.80	No	0	3	2

AUS, abdominal ultrasound; Hz, hertz; HRF, high risk findings; kg, kilogram.

### Impact of transducer frequency

**Figure 3:**
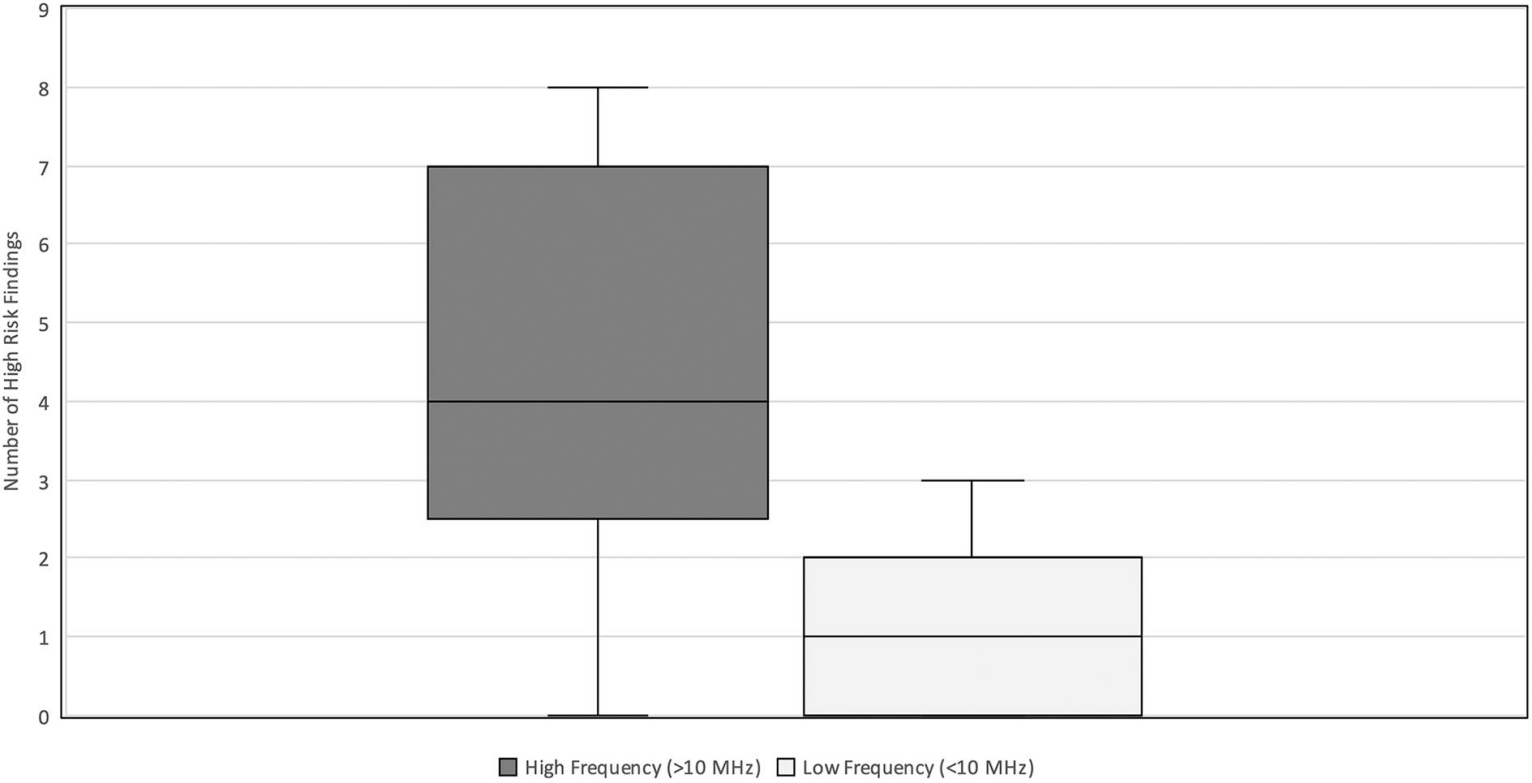
High-risk findings vs. Transducer Frequency. Relationship between the number of HRF commented on and the transducer frequency (MHz) used during neonatal abdominal ultrasound exams. The high frequency (>10 MHz) group had a median = 4 and IQR 3–7. The low frequency (≤10 MHz) group had a median = 1 and IQR-2. HRF, high risk findings; IQR, inter-quartile range.

**Figure 4:**
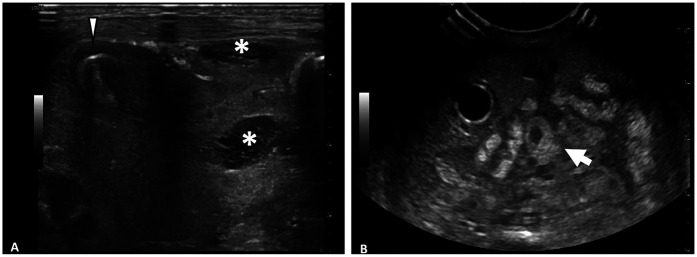
Comparison of abdominal ultrasound images obtained with high frequency vs. low frequency ultrasound transducer. **(A)** Image obtained with 6–15 MHz linear transducer. Improvd spatial resolution of 6–15 MHz transducer allows for visualization of fine details such as septations within fluid collections (*) and layers of intestinal wall (arrowhead). **(B)** Image obtained with 3–10 MHz transducer. Echogenic loop of bowel (arrow) seen, however further evaluation of intestinal wall limited by decreased spatial resolution.

The types of ultrasound transducers used included low frequency 3–10 MHz micro-convex, low frequency 2–9 MHz linear, high frequency 6–15 MHz linear, high frequency 8–18 MHz linear, and high frequency 4–20 MHz linear. Ultrasounds performed with linear, high-frequency transducers (>10 MHz) yielded higher numbers of HRF (median = 4, IQR 3–7) compared to those performed with lower-frequency transducers (≤10 MHz) (median = 1, IQR 0–2) (*p* < 0.001) (Figure 3). The AUS obtained with the higher frequency transducer had a higher resolution of intestinal wall architecture than with the lower frequency transducer, as shown in Figure 4.

**Figure 5:**
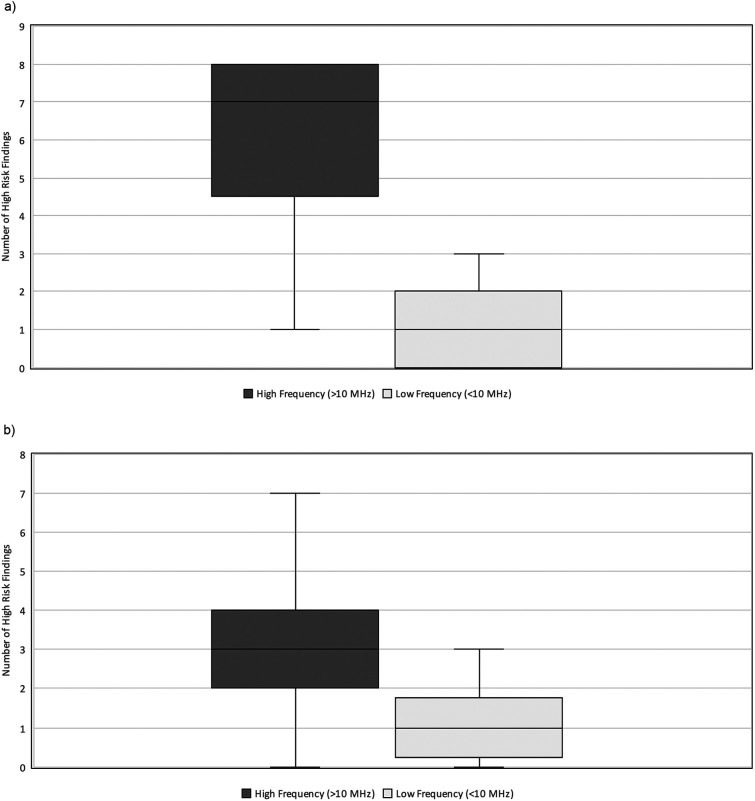
**(a)** High-risk findings vs. Transducer frequency (HZ) stratified by weight. Relationship between the number of HRF commented on and the transducer frequency used (MHz) during neonatal abdominal ultrasound exams in neonates weighing less than 2 kg at the time of exam. The high frequency (>10 MHz) group had a median = 7 and IQR 5–8. The low frequency (≤10 MHz) group had a median = 1 and IQR 0–2. HRF, high risk findings; IQR, inter-quartile range. **(b)** HRF vs. Transducer frequency (Hz) stratified by weight. Relationship between the number of HRF commented on and the transducer frequency used (MHz) during neonatal abdominal ultrasound exams in neonates weighing more than 2 kg at the time of exam. The high frequency (>10 MHz) group had a median = 3 and IQR 2–4. The low frequency (≤10 MHz) group had a median = 1 and IQR 0–2. HRF, high risk findings; IQR, inter-quartile range.

When stratified by patient weight, the impact of transducer frequency varied significantly. Neonates weighing <2 kg demonstrated a relatively greater difference in interpretability in exams performed with high vs. low-frequency transducers (median = 7, IQR 5–8 vs. median = 1, IQR 0–2, *p* = 0.005) (Figure 5A). Comparatively, in neonates weighing ≥2 kg, there was a relatively lower difference in interpretability (median = 3, IQR 2–4 vs. median = 1, IQR 0–2) (Figure 5B). Most notably, two neonates weighing 0.5 kg, who died with the surgical confirmation of SIP, had no HRF noted on AUS when a low-frequency transducer was used.

### Serial vs. single ultrasound examinations

**Figure 6:**
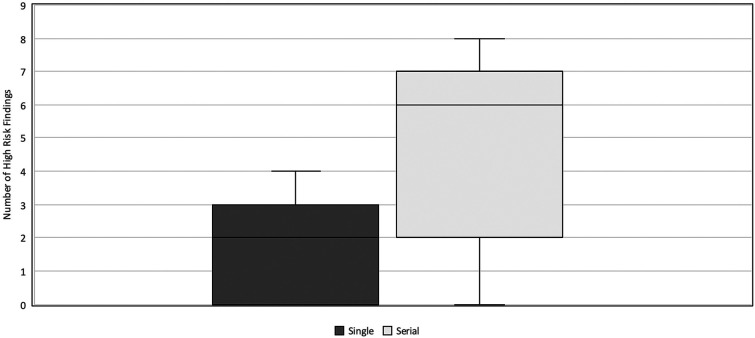
HRF in serial vs. single AUS. Comparison of the number of HRF commented on in AUS exams performed as a part of a set of serial examinations vs. those performed as single exam prior to surgery. The single AUS exam group had a median HRF = 2 and IQR 0–3. The serial AUS exam group had a median HRF = 6 and IQR 2–7. HRF, high risk findings; AUS, abdominal ultrasound; IQR, inter-quartile range.

Six out of 18 neonates underwent two to three AUS examinations within 7 days prior to exploratory laparotomy. AUS exams performed as part of the serial imaging protocol had a higher interpretability (median HRF = 6, IQR 2–7) compared to the single AUS exam (median HRF = 2, IQR 0–3) (*p* = 0.034) (Figure 6).

### Clinical characteristics

Neonates with clinical signs (hypotension or abdominal discoloration) demonstrated a slightly higher interpretability (median HRF = 3, IQR 1–7) compared to those without these clinical signs (median HRF = 2, IQR 1–3). Even in the absence of clinical signs or documented HRF, bowel perforation was confirmed in those infants at surgery.

### Effect of order quality

Examinations ordered with comprehensive and specific clinical details and indication for abdomen ultrasonography provided to the sonographers and radiologists had slightly higher interpretability (median HRF = 3, IQR 2–7) compared to those with less comprehensive indications (median HRF = 2, IQR 0–4). An example of a low-quality with less comprehensive order indication was “Evaluation for NEC”, whereas a high-quality order indication example was “Preterm neonate with abdominal discoloration, hypotension, and right lower quadrant fullness, evaluate all four bowel quadrants for intestinal ischemia”.

## Discussion

This is the first case series describing the clinical and technical factors affecting AUS imaging interpretability. Although the sample size is small in our pilot study, these findings provide valuable guidance for optimizing our AUS protocol and implementation process in our institution. Our study findings demonstrate a probable relationship between higher transducer frequency (>10 MHz) and increased interpretability. This trend was stronger among neonates weighing less than 2 kg. This can be explained from the fact that high-frequency transducers provided greater resolution, facilitating clearer visualization of subtle yet clinically significant findings such as bowel wall abnormalities and complex free fluid. Conversely, lower-frequency transducers (<10 MHz) yielded fewer HRF, likely due to reduced spatial resolution. This is consistent with previous literature supporting the use of ultra-high frequency transducer selection in this patient population (11). However, there is no current consensus on the minimum transducer frequency that should be used (15–17). These observations suggest a benefit to preferentially utilizing ultra-high-resolution transducers, particularly in very small neonates.

Our study indicates that serial ultrasound examinations provide higher interpretability compared to single examinations. This result may reflect the dynamic progression of neonatal abdominal diseases, where repeated assessments improve the detection of evolving pathologic changes, enabling more timely and precise surgical decisions. Additionally, serial imaging would provide improved clinical context and baseline imaging for comparison to the reading radiologists. Incorporating serial ultrasound evaluation into clinical management algorithms, especially when initial findings are equivocal or clinical suspicion remains high, may enhance diagnostic certainty and patient outcomes.

Clinical presentation was another important factor influencing interpretability of ultrasound images. There are numerous clinical variables described in prediction of surgical NEC, however many of these variables are either non-specific or subjective. In our study, abdominal discoloration and refractory hypotension were chosen for their high specificity and relative lack of subjectivity (14, 23). Neonates exhibiting hypotension or abdominal discoloration had slightly higher numbers of explicitly documented HRF. This finding suggests that the interpretability of AUS may be enhanced when performed in patients with relevant clinical indications and a higher pre-test probability of intestinal pathology.

**Figure 7:**
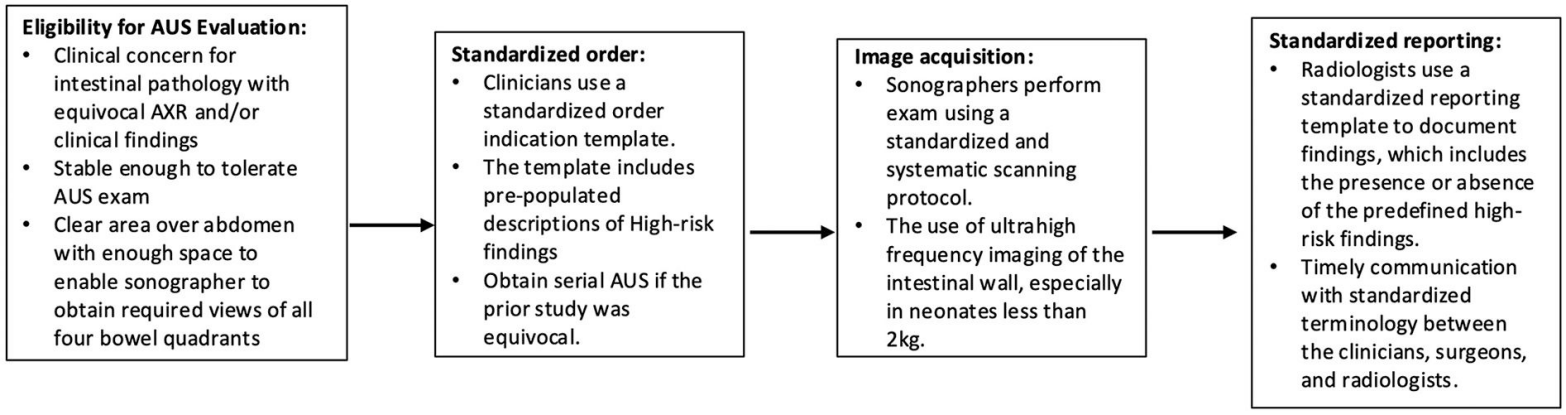
Proposed workflow for standardized ordering, scanning, and reporting of HRF in AUS. NEC, necrotizing enterocolitis; AUS, abdominal ultrasound; AXR, x-ray; HRF, high risk findings.

Although comprehensive and specific clinical order indications showed a trend toward improved interpretability, this association was not as strong as compared to transducer frequency and serial assessment. This still highlights an opportunity for further refinement in clinician-radiologist communication to enhance interpretation comprehensiveness. An example of this would be standardized order sets and reporting templates that would allow for consistent terminology and decreased variability in reporting (Figure 7). Such tools have been successfully utilized at other institutions (11, 24, 25).

In our experience, there was a gradual improvement in interpretability after standardizing the workflow. HRF were not documented for the first 4 neonates—thus no positive HRF were identified even in neonates who later had confirmed bowel perforation. This led to subsequent standardization of workflow and increased documentation of HRF in the subsequent patients.

We acknowledge several limitations of this study, including its retrospective design, relatively small sample size limiting detailed statistical analysis, non-independence from repeated measures, variability in AUS examinations performed by different technicians, and subjective differences in radiologists’ interpretations. Additionally, interpretability was quantified solely by the number of explicitly reported HRF, which may underestimate true clinical utility if certain findings were implicitly considered on the obtained images but not documented, or if certain ultrasound views were not acquired during the AUS. This quantitative methodology did not consider the weighting of each HRF. In practice, findings such as pneumoperitoneum would be weighted more heavily than other more subtle findings such as bowel wall thickening. Because of the retrospective study design, we were unable to determine the timing of AUS in relation to the surgical decision-making process. Despite these limitations, this pilot study provides valuable information for an ongoing quality improvement project in standardized AUS protocols in neonates with suspected intestinal injury.

## Conclusion

Using high-frequency transducers, particularly for scanning neonates weighing less than 2 kg, and incorporating serial examinations into diagnostic workflows has the potential to improve interpretability and diagnostic accuracy of AUS in neonates with intestinal injury. Providing clinical details, clearly stating the indications on AUS order, and utilizing standardized interpretation reports may further improve communication between clinicians and radiologists.

## Data Availability

The original contributions presented in the study are included in the article/Supplementary Material, further inquiries can be directed to the corresponding author/s.
